# Optimal buprenorphine exposure to treat patients with opioid use disorder in the era of fentanyl and polysubstance use

**DOI:** 10.3389/fphar.2026.1839067

**Published:** 2026-09-08

**Authors:** L. R. L. Lohmer, M. K. Greenwald, R. Kakar, C. M. Laffont

**Affiliations:** 1 Research and Development, Indivior Pharmaceuticals Inc., North Chesterfield, VA, United States; 2 Department of Psychiatry and Behavioral Neurosciences, Wayne State University School of Medicine, Detroit, MI, United States; 3 Segal Trials Miami Lakes Early Phase and Inpatient Site, Miami Lakes, FL, United States

**Keywords:** (extended-release) buprenorphine, exposure-response, fentanyl and stimulant co-use, opioid use disorder, optimized treatment outcomes

## Abstract

**Background:**

In recent years, the United States opioid epidemic has been characterized by rising polysubstance use and increasing deaths due to fentanyl overdose, with stimulants playing a key role. We conducted an integrated analysis to evaluate the impact of fentanyl and polysubstance use on buprenorphine exposure-response relationships and to assess whether higher buprenorphine exposures may be required.

**Methods:**

Data from two large clinical trials of extended-release buprenorphine (BUP-XR) conducted before and after the widespread emergence of fentanyl in patients with moderate-to-severe opioid use disorder were integrated for analysis (N = 989). Approximately half of participants in the most recent study reported daily fentanyl use at baseline, with 29.2% reporting co-use of amphetamines/methamphetamines. Non-linear mixed effects models were developed to characterize treatment effects on opioid use and opioid craving. Time-to-event modeling was applied to evaluate treatment retention.

**Results:**

While sustained buprenorphine plasma concentrations of 2–3 ng/mL were adequate for most patients, higher concentrations of 5–6 ng/mL conferred additional benefits in specific subgroups, particularly among patients reporting daily fentanyl use in combination with amphetamines/methamphetamines. Additionally, patients from the most recent study required longer treatment duration to achieve maximum effects on opioid abstinence (6 months on average, although associated with large inter-patient variability). Frequent fentanyl use (≥2 times per day) was associated with higher levels of craving, especially among patients reporting injection as their primary route of administration. High craving emerged as a key determinant of treatment discontinuation, with a twofold higher dropout rate when craving >20 on a 100-mm visual analog scale. Older age and Black/African American race were associated with a lower dropout risk.

**Conclusion:**

Altogether, these analyses provide important insights into optimal buprenorphine exposure targets and advance understanding of patient treatment journey in the era of synthetic opioids and stimulant co-use.

## 1 Introduction

Despite recent declines in opioid overdose deaths, the United States (US) opioid crisis remains a major public health concern, characterized by evolving patterns in opioid use over time. Four distinct waves have been described over the past 2 decades (Friedman and Shover, 2023). The first wave of the opioid epidemic started in the late 1990s/early 2000s with a rise in overdose fatalities involving prescription opioids. The second wave began in 2010 driven by a shift to heroin, and the third wave started around 2013 with the emergence of high-potency synthetic opioids, predominantly illicitly manufactured fentanyl. Data now indicate a ‘fourth wave’ of the US overdose crisis in the face of rapidly rising polysubstance overdose deaths involving illicitly manufactured fentanyl, with stimulants (amphetamines/methamphetamines and cocaine) playing a key role (Han et al., 2021; Hedegaard et al., 2021; Friedman and Shover, 2023; Mills et al., 2026). Hence, while opioid overdose deaths are declining, fentanyl (and other synthetic opioids), stimulants plus opioids, and polysubstance use remain central drivers of overdose mortality.

Emerging evidence also suggests that concurrent use of opioids and stimulants (Coles et al., 2025), notably amphetamines/methamphetamines (Frost et al., 2021; Russell et al., 2023), may negatively impact receipt of medications for opioid use disorder (OUD), retention in treatment, and opioid abstinence during treatment. Patients may intentionally co-use stimulants to counteract the sedative effects of opioids, offset cognitive deficits, maintain alertness for safety, achieve a synergistic drug “high,” or endure opioid withdrawal symptoms (Ellis et al., 2018; Ivsins et al., 2022; Friedman and Shover, 2023). Others believe that methamphetamine can prevent or reverse opioid overdose (Ondocsin et al., 2023). Co-use can also be unintentional due to adulterated drug supply. Patterns of stimulant co-use (cocaine versus amphetamines/methamphetamines) appear to differ by geographic location (Friedman and Shover, 2023).

In this work, we present an integrated analysis of two large clinical trials of extended-release buprenorphine (BUP-XR; SUBLOCADE®) conducted before and after the widespread emergence of fentanyl in the illicit drug supply. This analysis was designed to address the significant shift in opioid-use patterns in the US with the rise of fentanyl and polysubstance use, particularly the co-use of opioids and stimulants. Our primary objective was to evaluate whether the relationship between buprenorphine plasma exposure and key efficacy outcomes–including opioid use, opioid craving, and treatment retention–has changed. Specifically, we sought to identify patient subgroups who might benefit from higher buprenorphine exposure to achieve optimal clinical outcomes.

## 2 Materials and methods

The analysis included data from two large clinical trials whose primary objective was to assess the efficacy of low (100 mg) and high (300 mg) maintenance doses of BUP-XR. Study 1 (NCT02357901) was the pivotal Phase 3 efficacy study that supported the regulatory approval of BUP-XR. Study 2 (NCT04995029) was conducted post approval to further evaluate patient subpopulations that may benefit from the 300-mg maintenance dose. While Study 1 was conducted in 2015–2016 when fentanyl use was not prevalent, Study 2 was conducted more recently in 2021–2024 during high fentanyl prevalence. Both studies included measurements of buprenorphine plasma concentrations, urine drug screens, self-reports of opioid use, and opioid craving (assessed using the same visual analogue scale), enabling an integrated analysis.

### 2.1 Study 1

Study 1 (summarized in Supplementary Table 1) was a randomized, double-blind, placebo-controlled study conducted at 36 treatment centers in the US between January 28, 2015, and April 29, 2016. Eligible participants were treatment-seeking individuals at least 18 years old, meeting Diagnostic and Statistical Manual of Mental Disorders, Fifth Edition (DSM-5) criteria for moderate to severe OUD.

Participants were first inducted and dose-stabilized on transmucosal buprenorphine over 1–2 weeks to reach daily buprenorphine doses between 8 mg and 24 mg. Eligible participants were randomized 2:2:1 on Day 1 to receive injections every 4 weeks: either 6 injections of 300 mg BUP-XR (300/300 mg dosing regimen); 2 injections of 300 mg BUP-XR followed by 4 injections of 100 mg BUP-XR (300/100 mg dosing regimen); or 6 injections of placebo (volume-matched for 100 mg or 300 mg injections of BUP-XR). After the first injection of BUP-XR or placebo on Day 1, no supplemental buprenorphine was allowed during the study, except for a 5-day taper (6–2 mg/day) on Days 1–5. All participants received weekly individual drug counseling throughout the study. Additional details on the study design are available in Haight et al. (2019).

Centrally tested urine drug screens and self-reports documenting past-week drug use were assessed at screening and every week after each injection of BUP-XR or placebo; a urine drug screen was also performed 24 h after each BUP-XR injection. Substances assessed by urine drug screen included opioids, oxycodone, methadone, buprenorphine (screening only), cocaine, amphetamines/methamphetamines, cannabinoids, barbiturates, benzodiazepines, and phencyclidine. Fentanyl was not specifically assessed given the low prevalence of fentanyl at the time and the unavailability of routine fentanyl screening. Self-reports of past-week drug use utilized the Timeline Followback interview whereby participants reported use or no use; frequency and amount of use were not captured. Participants also rated their current craving severity using a 0–100-mm Opioid Craving Visual Analog Scale (OC-VAS) where zero meant “no craving at all” and 100 meant “strongest craving ever”. The OC-VAS was validated psychometrically on a large OUD patient population, supporting its use to measure craving severity and its ability to predict opioid use (Boyett et al., 2021). Finally, opioid withdrawal severity was quantified using the Clinical Opiate Withdrawal Scale (COWS; Wesson and Ling, 2003) and Subjective Opioid Withdrawal Scale (SOWS; Handelsman et al., 1987). Scores on the OC-VAS, COWS, and SOWS were obtained at the same times as urine drug screens, with additional measurement performed during induction and stabilization with transmucosal buprenorphine prior to randomization to BUP-XR or placebo.

Blood samples to determine buprenorphine plasma concentration were taken on the last day of transmucosal buprenorphine treatment (Day −1: pre-dose and 1–2 h post-dose) and during the randomized treatment phase (pre-dose and every week after each injection, with additional samples at 4 and 24 h post-dose). Plasma concentrations of buprenorphine were determined using a previously validated liquid chromatography and tandem mass spectrometry (LC-MS/MS) method with a lower limit of quantitation of 0.050 ng/mL (Nasser et al., 2016). A blood sample was collected before the first BUP-XR or placebo injection on Day 1 to assess various single nucleotide polymorphisms (SNPs) for opioid receptor subtypes (including rs678849 for the δ-opioid receptor gene, *OPRD1*) and dopamine D_2_ receptor.

### 2.2 Study 2

Study 2 (summarized in Supplementary Table 1) was a randomized, double-blind study conducted at 28 treatment centers in the US and Canada between October 26, 2021, and June 26, 2024. Eligible participants were treatment-seeking individuals at least 18 years old, meeting criteria for moderate or severe OUD (based on DSM-5) for 90 days or more prior to informed consent, and meeting criteria for high-risk opioid use. High-risk opioid use was defined as using opioids an average of ≥5 days per week during the last 4 weeks via the injection route, or by any route at quantities ≥500 mg intravenous heroin equivalent, or any dose of highly potent synthetic opioids (excluding fentanyl transdermal patches).

In this study, patients were initially randomized 2:1 to receive rapid induction (single 4-mg transmucosal buprenorphine dose administered 1 h before BUP-XR) or standard induction (≥7 days on transmucosal buprenorphine), followed by two injections of 300 mg BUP-XR administered 1 week apart on Week 1 (Day 1) and Week 2 (Days 8–12). On Week 6 (4 weeks after the second injection), participants were randomized 1:1 to receive 8 additional maintenances doses of either 100 mg or 300 mg of BUP-XR every 4 weeks. Additional details on induction and maintenance are provided in Shiwach et al. (2025a) and Shiwach et al. (2025b), respectively. Participants were not permitted to use supplemental transmucosal buprenorphine after Day 1. All participants received counseling throughout the study.

Participants returned to the site weekly for the first 10 weeks, at each injection visit from Weeks 10–34, on Weeks 36 and 38, and at random visits from Injection four through Injection 10 (2 weeks post-injection ±7 days). At each visit, participants completed a urine drug screen and a Timeline Followback documenting past-week drug use. Unlike Study 1, the frequency of opioid use was captured during the Timeline Followback interview. Point-of-care urine drug screen were performed at screening, on induction day, prior to BUP-XR injection on Week 1 Day 1 (if different from induction day), and at Week 6. Centrally tested urine drug screens were performed at all other visits. Substances assessed by urine drug screen included opioids, fentanyl, oxycodone, morphine, methadone, buprenorphine (screening and induction only), and other substances of abuse like those evaluated in Study 1. At all visits except random visits, participants rated their current craving and worst past-week craving severity using the same OC-VAS as in Study 1, and their opioid withdrawal severity was quantified using the COWS.

Blood samples to determine buprenorphine plasma concentration were obtained before and 2 h following the first injection on Week 1, weekly through Week 10, and then every 4 weeks through Week 38 (including pre-dose samples) with another sample collected on Week 36. Plasma samples were analyzed for buprenorphine using the same validated LC-MS/MS method as in Study 1. A blood sample was also collected before the first BUP-XR injection on Day 1 for DNA analysis to assess *OPRD1* SNP rs678849 genotype variation.

### 2.3 Modeling efficacy outcomes

Three models characterizing opioid use, opioid craving, and treatment retention were developed and integrated into an overall exposure-response model.

Existing population pharmacokinetic models for transmucosal buprenorphine and BUP-XR (Jones et al., 2021) were fitted to buprenorphine plasma concentration data from both studies to generate predictions of individual pharmacokinetic parameters (Empirical Bayes estimates [EBEs]) that were further used in exposure-response analyses. As described in Jones et al. (2021), the BUP-XR population pharmacokinetic model was built from 19,686 observations collected in 570 subjects and was very robust in describing the pharmacokinetics of buprenorphine across 3 BUP-XR studies (including Study 1) and multiple injections (up to 12 monthly injections covering a 1-year exposure). Only participants with at least 1 quantifiable buprenorphine plasma concentration post BUP-XR were included in the pharmacokinetic analysis.

Opioid use status for the past week was analyzed as a binary variable whose value was either 0 (no opioid use) or one (opioid use). A value of 0 required both urine drug screen and self-report to be negative for opioids at the same visit. Missing data were not imputed. The probability of negative opioid use was modeled using mixed-effects logistic regression. An E_max_ relationship with buprenorphine plasma concentration was evaluated based on observed data and prior knowledge on buprenorphine exposure-response and receptor occupancy analyses. Only participants who had at least one post-BUP-XR measure of opioid use and pharmacokinetic data were included in the analysis.

Current craving OC-VAS scores were categorized as 0, 1–5, 6–20 and >20 mm for the analysis (categorization was data-driven and due to the variability in the data). Craving >20 (category 4) was considered as clinically significant (Rosenthal et al., 2013). Categorical opioid craving data were analyzed using a mixed-effects logistic regression model for ordinal data. An E_max_ relationship with buprenorphine plasma concentration was evaluated based on observed data, prior knowledge, and model fit. Only participants with at least one craving observation post-BUP-XR and pharmacokinetic data were included in the analysis.

Finally, a time-to-event model was developed to describe treatment retention in both studies and to assess the relationship with measures of efficacy (opioid use and opioid craving). Different distribution functions (constant hazard, Gompertz, Weibull) were tested for the hazard of dropout. As further described, dropout was successfully predicted from participant characteristics at baseline as well as observed measures of efficacy, supporting missing-at-random mechanisms. Therefore, each model for opioid use, opioid craving, and treatment retention could be fitted separately, and the combined (integrated) model was used for model evaluation. The analysis population was the same as the pharmacokinetic population.

Covariate analyses were conducted to assess the impact of participants’ characteristics and other factors on opioid use, craving, and dropout rate. Post-hoc statistical analyses of Study 2 (described in Shiwach et al., 2025b) demonstrated that participants with frequent fentanyl use at baseline (i.e., using fentanyl daily and/or ≥14 times/week) had better treatment responses with the 300-mg maintenance dose of BUP-XR than with the 100-mg maintenance dose, indicating that higher buprenorphine plasma concentrations might be needed in this subpopulation. Frequent fentanyl use (daily, ≥2 times/day, ≥14 times/week) was therefore tested as a covariate in all models. Other covariates evaluated included demographics (age, weight, body mass index, sex, race), *ORPD1* rs678849 genotype (identified as significant in prior analyses), and employment status at baseline, as well as baseline substance use information (use of injection as main route of use, stimulant use [cocaine, amphetamines/methamphetamines], cannabis use, and polysubstance use). Polysubstance use was defined as the number of substances in addition to opioids that were reported at baseline. For the dropout model, additional covariates tested were the observed measures of opioid use and opioid craving handled as time-varying covariates using the last-observation carried-forward (LOCF) approach. With this approach, the whole history of each participant’s craving records or opioid use records from the beginning of the trial was evaluated to predict survival.

Covariates were first assessed based on exploratory Kaplan-Meier plots for treatment retention, or plots of observations stratified by covariates or EBEs versus covariates (base model without covariates) for opioid use and craving models. The identification of potential relationships with EBEs was based on visual inspection, statistical testing (Kruskal–Wallis rank sum test for categorical covariates and linear/non-linear regression [power model] for continuous covariates using a significance level [α] of 0.05), and physiological relevance. Covariate relationships identified, as well as those previously found to be significant in previous exposure-response analyses (Laffont et al., 2022), were further tested using forward/backward selection and significance levels of 0.05 and 0.001, respectively.

Model evaluation was performed using visual predictive checks (Bergstrand et al., 2011) comparing observations to model predictions generated under the same study design.

### 2.4 Software

All exploratory data analyses, construction of analysis datasets, and presentations of data were performed using R software (version 4.3.3 and higher). Models were developed in Pumas AI version 2.6.1 (Pumas-AI Inc., Dover, DE), using either first-order conditional estimation (FOCE) or Laplace with interaction (LaplaceI) estimation method. Visual predictive checks were generated in Pumas AI or R based on model simulations.

### 2.5 Study approval

Both studies were conducted in accordance with principles and requirements of the International Council for Harmonization Good Clinical Practice guidelines and the principles of the Declaration of Helsinki. Written informed consent was obtained prior to any study-related procedures. Clinical study protocols, informed consent forms, and all other appropriate study-related documents were reviewed and approved by Institutional Review Boards.

## 3 Results

### 3.1 Patient characteristics

Key baseline characteristics of patients included in the analysis are summarized per study in Table 1. Participants were predominantly male (66.3% and 58.0% in Studies 1 and 2, respectively). The high-risk population from Study 2 included 49.4% of participants reporting daily fentanyl use at baseline; 44.0% of participants reported using fentanyl ≥2 times/day. Fentanyl use was not specifically assessed in Study 1 (conducted in 2015–2016) and was assumed to be minimal due to low population prevalence at that time; thus, frequent fentanyl use (daily, ≥2 times/day) was imputed as absent in Study 1.

**TABLE 1:** Key baseline characteristics for patients included in the analysis.

Patient characteristics	Study 1 (2015–2016, n = 489)	Study 2 (2021–2024, n = 500)
Race		
White	349 (71.3%)	367 (73.4%)
Black/African american	130 (26.6%)	104 (20.8%)
Others	10 (2.0%)	29 (5.8%)
Age (years)		
Mean (SD)	39.7 (11.1)	41.2 (10.6)
Median [min, max]	38 [19, 64]	40 [18, 72]
Sex		
Male	324 (66.3%)	290 (58.0%)
Employment		
Yes	165 (33.7%)	197 (39.4%)
Missing	26 (5.3%)	0 (0%)
DSM-5 severity		
Moderate	147 (30.1%)	55 (11.0%)
Severe	337 (68.9%)	445 (89.0%)
Missing	5 (1.0%)	0 (0%)
Main route of administration		
Injection	214 (43.8%)	146 (29.2%)
Smoking	32 (6.5%)	206 (41.2%)
Snorting	125 (25.6%)	134 (26.8%)
Oral	118 (24.1%)	13 (2.6%)
Other	0 (0.0%)	1 (0.2%)
Daily fentanyl		
Yes	0 (0.0%)^a^	247 (49.4%)
Fentanyl ≥2x/day		
Yes	0 (0.0%)^a^	220 (44.0%)
Fentanyl ≥14x/week		
Yes	0 (0.0%)^a^	223 (44.6%)
Cocaine		
Yes	212 (43.4%)	129 (25.8%)
Amphetamines/Methamphetamines		
Yes	97 (19.8%)	300 (60.0%)
Cannabis		
Yes	251 (51.3%)	297 (59.4%)
Polysubstance use		
At least one	346 (70.8%)	474 (94.8%)
Mean (SD)	1.29 (1.14)	2.54 (1.29)
Median [min, max]	1 [0, 5]	3 [0, 6]
Daily fentanyl + amphetamines/Methamphetamines		
Yes	0 (0%)	146 (29.2%)
Fentanyl ≥2x/day + injection use		
Yes	0 (0%)	30 (6.0%)
*OPRD1* genotype		
CC	337 (34.1%)	170 (34%)
CT	426 (43.1%)	221 (44.2%)
TT	178 (18.0%)	88 (17.6%)
Missing	48 (4.85%)	21 (4.2%)

Includes depression, anxiety, bipolar disorder, and schizophrenia.

^a^ Imputed as fentanyl was not specifically evaluated in this study.

Compared to the population in Study 1, participants in Study 2 had more polysubstance use (94.8% versus 70.8%), 41% less cocaine use (25.8% versus 43.4%), and 3-fold higher amphetamine/methamphetamine use (60.0% versus 19.8%). Also, fewer participants in Study 2 reported injection as their main route of use (29.2% versus 43.8% in Study 1). Notably, 50.6% of participants with daily fentanyl use reported smoking as their main route of use, compared to 27.1% snorting and 19.0% injection.

### 3.2 Buprenorphine plasma concentrations

In total, 18,400 buprenorphine plasma concentrations (11,362 from 489 participants in Study 1 and 7,038 from 582 participants in Study 2) were available for analysis. Only 1.1% of concentrations were below the limit of quantification, mostly after transmucosal buprenorphine administration, and were excluded. The existing population pharmacokinetic models for BUP-XR and transmucosal buprenorphine provided an adequate description of buprenorphine concentration-time data for both treatment groups in Study 2 (Supplementary Figure 1). Model evaluation for Study 1 was conducted as part of previous modeling work, and visual predicted checks are available in Jones et al. (2021). Individual pharmacokinetic parameters (EBEs) were derived for both studies and used for exposure-response analyses of opioid use and opioid craving data.

### 3.3 Opioid use model

**FIGURE 1:**
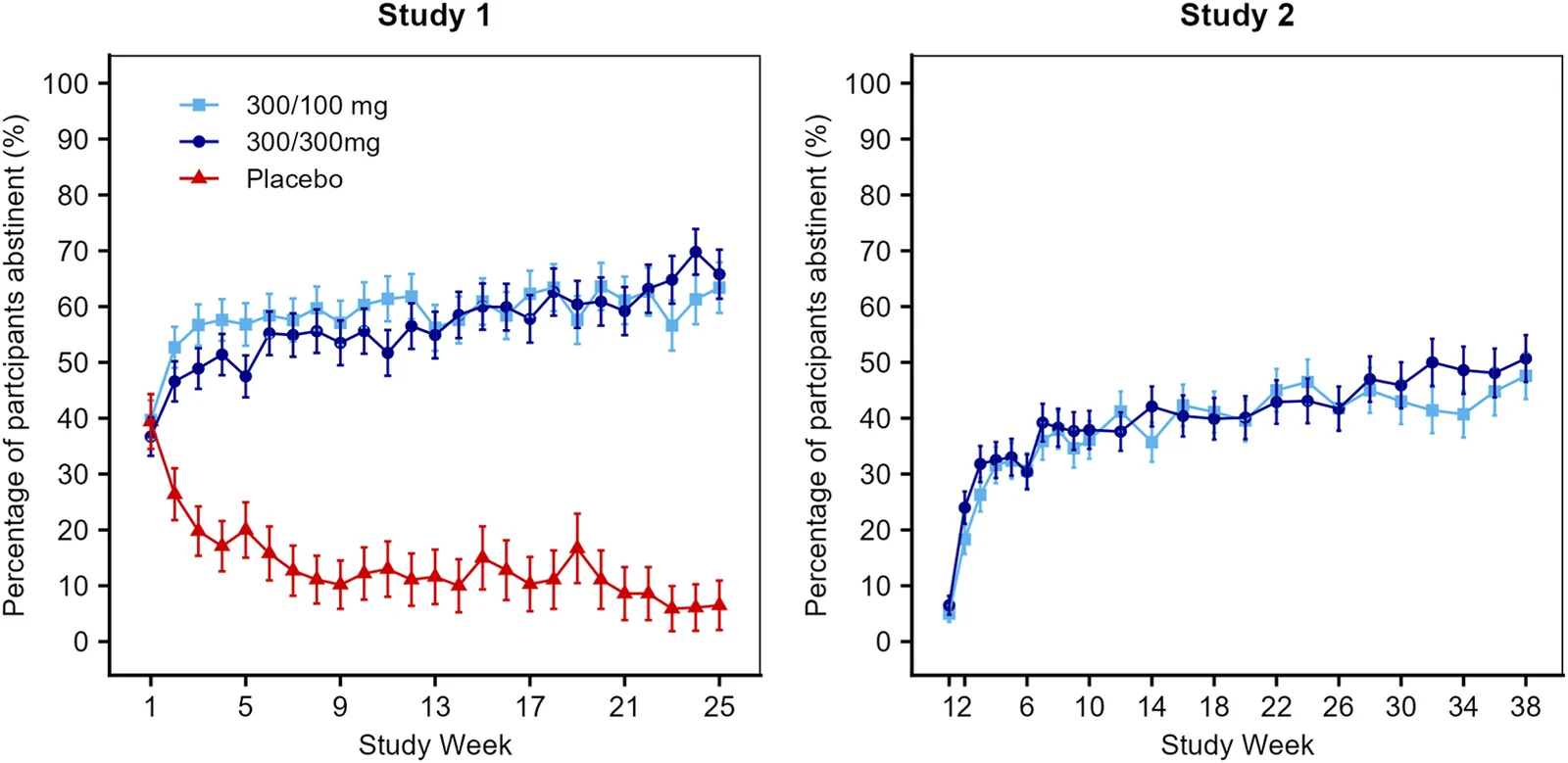
Proportion of Participants Abstinent over Time by Treatment Arm in Study 1 and Study 2. Open-label data from participants not randomized to an extended-release buprenorphine (BUP-XR) maintenance dose on Week 6 were excluded from Study 2 plot. Errors bars indicate standard errors.

A total of 19,811 observations (10,943 in Study 1 and 8,868 in Study 2) obtained in 989 patients (489 in Study 1 and 500 in Study 2) were used to build the exposure-response model for opioid use. Opioid use data, shown in Figure 1, were well described with a mixed-effects logistic regression model including an E_max_ relationship to describe the effect of buprenorphine plasma concentration. In prior Study 1 analysis (Laffont et al., 2022), a specific intercept was estimated for the 300/100 mg treatment arm relative to the 300/300 mg treatment arm. This “100-mg arm” effect in Study 1 was incorporated into the base model for the present analysis. Maximum treatment response and severity of disease differed between studies; thus, separate parameters for E_max_ and intercepts were estimated per study.

**FIGURE 2:**
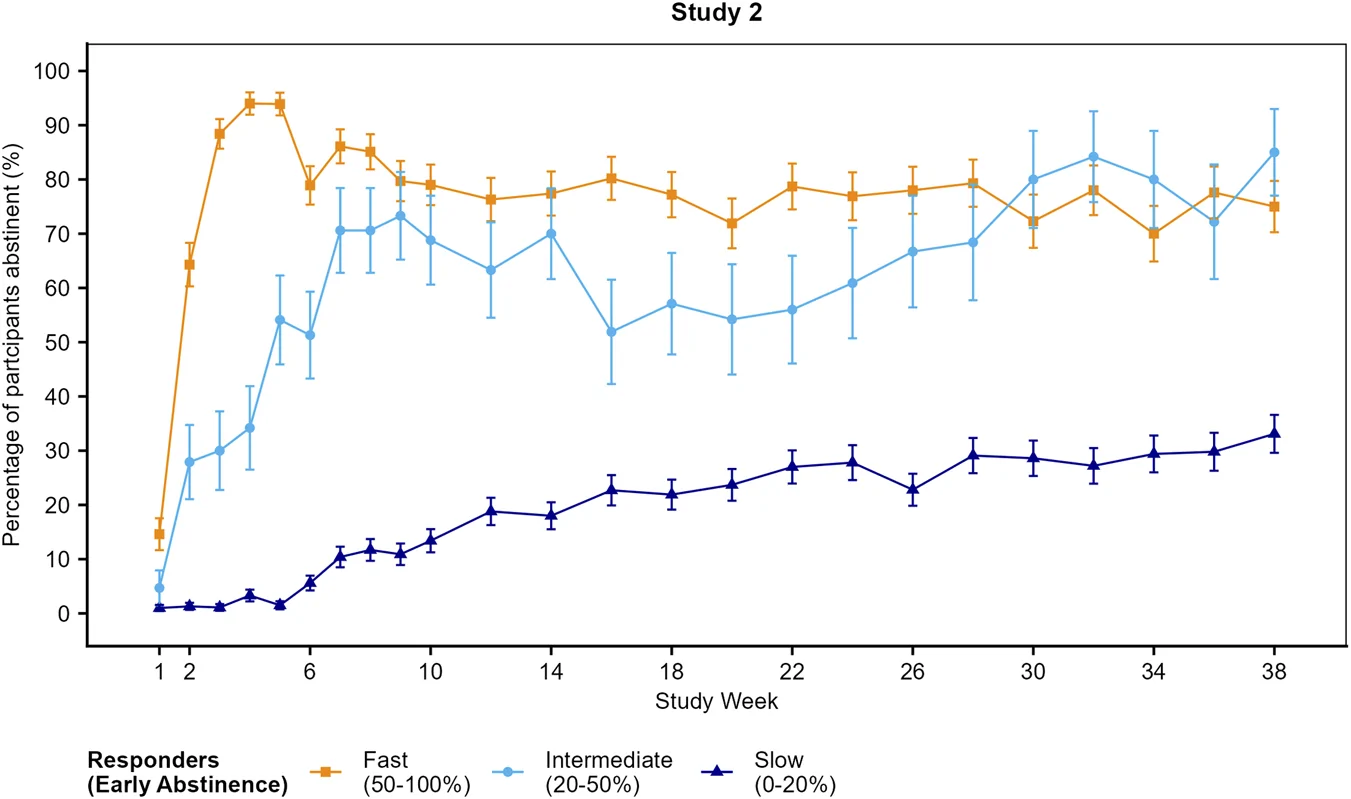
Time Course of Opioid Abstinence in Study 2 in Subpopulations Defined as Slow, Intermediate, or Fast Responders. Participants were categorized as slow, intermediate, or fast responders based on early abstinence calculations over the first two dosing intervals in Study 2 (Weeks 2 to 6). Fast responders were qualified as those having an early abstinence >50% (n = 145, 29%), intermediate responders as those having an early abstinence >20% and ≤50% (n = 43, 9%), and slow responders as those having an early abstinence ≤20% (n = 312, 62%). Errors bars indicate standard errors.

Participants in Study 2 also required longer time on treatment to achieve maximum response. We estimated percentages of fast and slow responders based on calculations of early abstinence over the first two dosing intervals, with slow response defined as early abstinence ≤20% and fast response defined as early abstinence >50%. About two-thirds (62.4%) of participants in Study 2 were slow responders (vs. 30.3% in Study 1) and only 29.0% were fast responders (vs. 53.0% in Study 1). The higher portion of slow-responders in Study 2 required including an effect compartment model specific to Study 2, with a k_e0_ estimated at 0.000856 h^-1^ corresponding to a half-life of approximately 34 days (approximately 6 months to achieve maximum response). This value was associated with large inter-patient variability (143%). The time course of opioid abstinence for slow and fast responders in Study 2 is illustrated in Figure 2.

**FIGURE 3:**
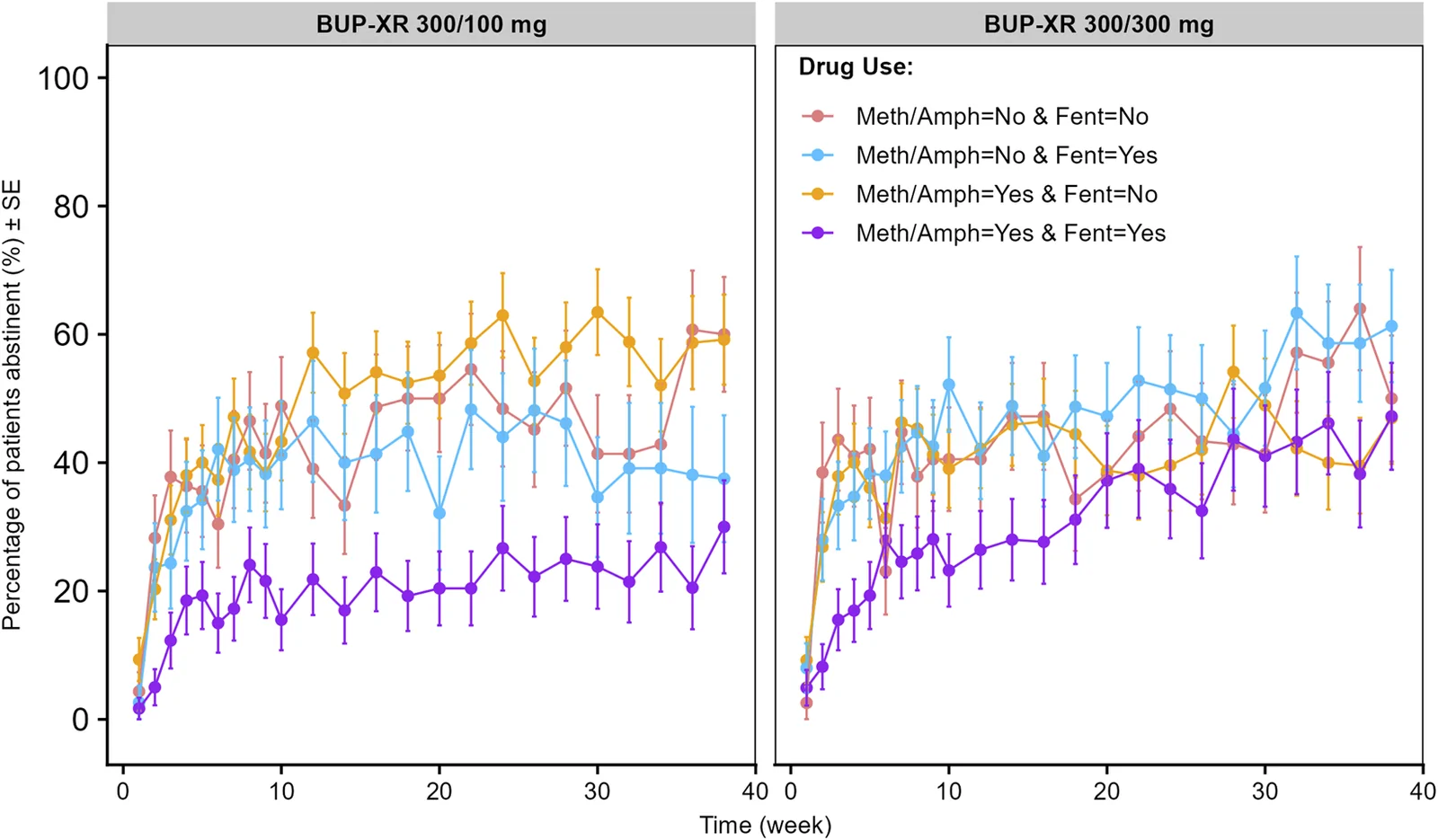
Proportion of Patients Abstinent in Study 2 for BUP-XR 300/100 mg and BUP-XR 300/300 mg Treatment Arms. Participants within each treatment arm were stratified by methamphetamine/amphetamine (Meth/Amph) and daily fentanyl use (Fent) reported at baseline. Approximately 21% (n = 46) and 18% (n = 39) of participants reported no baseline methamphetamine/amphetamine or daily fentanyl use (red line) for the 300/100 mg and 300/300 mg treatment arms, respectively; 17% (n = 38) and 23% (n = 50) reported no baseline methamphetamine/amphetamine use but did report daily fentanyl use (blue line) for the 300/100 mg and 300/300 mg treatment arms, respectively; 34% (n = 75) and 31% (n = 67) reported baseline methamphetamine/amphetamine use but not daily fentanyl use (orange line) for the 300/100 mg and 300/300 mg treatment arms, respectively; and 27% (n = 60) and 28% (n = 61) reported both baseline methamphetamine/amphetamine and daily fentanyl use (purple line) for the 300/100 mg and 300/300 mg treatment arms, respectively. Errors bars indicate standard errors.

The covariate analysis pointed to several significant effects. A higher EC_50_ (buprenorphine concentration yielding half-maximal effect) was estimated for participants injecting opioids (1.74x higher EC_50_) and those reporting daily fentanyl use in combination with amphetamines/methamphetamines at baseline (8.25x higher EC_50_), indicating those patients might benefit from higher buprenorphine exposure. The effect of daily fentanyl use in combination with amphetamine/methamphetamine use was evident in exploratory analyses of abstinence outcomes as illustrated in Figure 3. Employment at baseline was associated with a higher probability of abstinence in the absence of any buprenorphine treatment, and E_max_ was 16% lower in Black/African American participants. Parameter estimates of the final model are presented in Table 2. The model accurately described observed measures of opioid use across studies for both active and placebo treatment groups (Supplementary Figure 2). Final model equations are included in the Supplemental Material.

**TABLE 2:** Parameter estimates for the final opioid use model.

Parameter	Description	Estimate (RSE%)
α Study 1	Intercept study 1	−4.28 (9.7)
α Study 2	Intercept study 2	−6.83 (7.6)
θ_α_, 100 mg, Study 1	Coefficient on α for 100 mg in study 1	0.764 (8.5)
θ_α_, employment	Coefficient on α for baseline employment	0.796 (6.8)
E_max_, Study 1	E_max_ for study 1	6.68 (6.7)
E_max_, Study 2	E_max_ for study 2	10.0 (3.8)
θ_Emax_, race	Coefficient on E_max_ in Black/AA	0.843 (4.8)
EC_50_	Concentration (ng/mL) reaching half of E_max_	0.999 (22.1)
θ_EC50_, injection	Coefficient on EC_50_ for injection use at baseline	1.74 (35.7)
θ_EC50_, fentanyl/amph	Coefficient on EC_50_ for daily fentanyl and amphetamine/methamphetamine co-use at baseline	8.25 (18.5)
k_e0_, Study 2	Equilibrium rate constant (study 2) (h^-1^)	0.000856 (13.3)
IIV α	SD	2.27 (13.2)
IIV EC_50_	CV%	171 (14.1)
IIV k_e0_	CV%	143 (8.8)

AA, African americans; CV%, Coefficient of variation expressed as a percentage; IIV, Interindividual variability; SD; standard deviation; RSE, Relative standard error. ‘Injection use’ means that injection was reported as the main route of opioid use at baseline.

### 3.4 Opioid craving model

A total of 18,093 observations (11,347 in Study 1 and 6,746 in Study 2) obtained in 987 participants (489 in Study 1 and 498 in Study 2) were used to build the opioid craving exposure-response model. The craving data were best described with a mixed-effects logistic regression model that included an E_max_ relationship with buprenorphine plasma concentration. Buprenorphine EC_50_ was estimated at 0.4 ng/mL, but this estimate was associated with high imprecision (66%). As for the opioid use model, a “100-mg arm” effect in Study 1 and separate parameters for E_max_ and intercepts per study were estimated. A placebo model was selected to better describe the placebo craving data from Study 1. The same equilibration rate k_e0_ (resulting in a half-life of 13 days) described the time to maximal placebo effect in Study 1 and the slower treatment response in Study 2.

**FIGURE 4:**
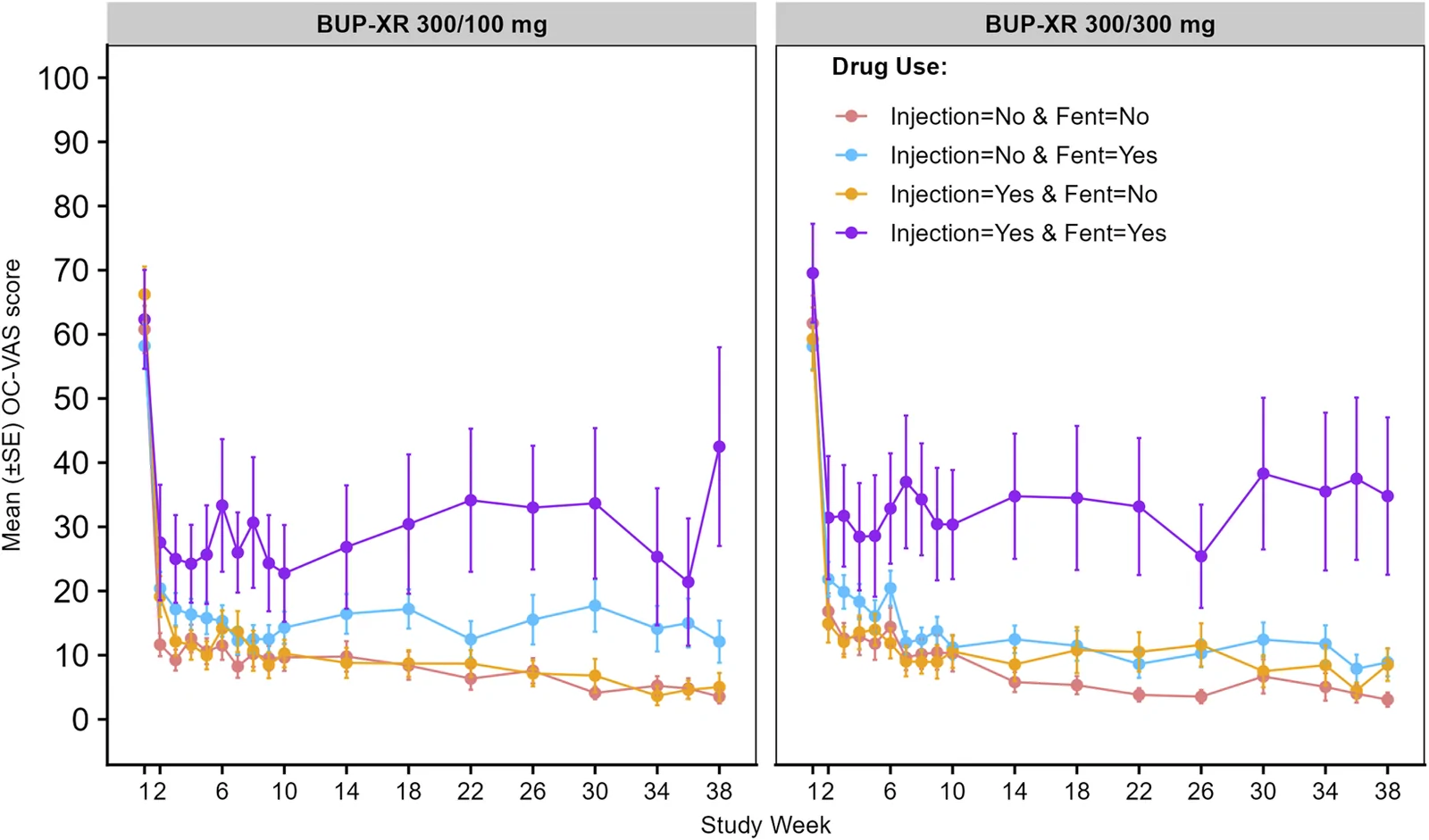
Mean Opioid Craving on Visual Analog Scale in Study 2 for BUP-XR 300/100 mg and 300/300 mg Treatment Arms. Participants within each treatment arm were stratified by frequent fentanyl use (≥2 times/day; Fent) and injection reported as the main route of opioid use at baseline. Approximately 35% (n = 75) and 31% (n = 68) of participants reported no baseline injection or frequent fentanyl use (red line) for the 300/100 mg and 300/300 mg treatment arms, respectively; 36% (n = 78) and 41% (n = 88) reported no baseline injection but did report frequent fentanyl use (blue line) for the 300/100 mg and 300/300 mg treatment arms, respectively; 26% (n = 57) and 22% (n = 47) reported baseline injection but not frequent fentanyl use (orange line) for the 300/100 mg and 300/300 mg treatment arms, respectively; 4% (n = 9) and 6% (n = 14) reported both baseline injection and frequent fentanyl use (purple line) for the 300/100 mg and 300/300 mg treatment arms, respectively. Errors bars indicate standard errors.

Covariate analysis identified several factors that contributed to craving outcomes. The maximum treatment response (E_max_) was estimated to be 26% lower in participants reporting frequent fentanyl use at baseline (≥2 times/day) and 80% lower in participants reporting both frequent fentanyl use *and* injection use at baseline. This aligns with observations in Study 2 showing higher craving scores in patients reporting both frequent fentanyl use and injection as the main route of use at baseline (6% of the Study 1 population; Figure 4). There was a 43% higher E_max_ in Black/African Americans, likely reflecting under-reporting of craving severity in this population. Parameter estimates of the final model are presented in Table 3. As shown by visual predictive checks in Supplementary Figure 3, the final model adequately fitted the data across studies and treatment groups. Final model equations are included in the Supplemental Material.

**TABLE 3:** Parameter estimates for the final opioid craving model.

Parameter	Description	Estimate (RSE%)
α_1_, Study 1	Intercept 1 for study 1	−5.39 (7.2)
α_1_, Study 2	Intercept 1 for study 2	−8.61 (16)
θ_α_ 100 mg, Study 1	Coefficient on α_1_ for 100 mg in study 1	0.971 (4.2)
δ_1_, Study 1	Delta between α_2_ and α_1_ in study 1	2.42 (4.3)
δ_1_, Study 2	Delta between α_2_ and α_1_ in study 2	3.30 (6.5)
δ_2_, Study 1	Delta between α_3_ and α_2_ in study 1	1.92 (4.9)
δ_2_, Study 2	Delta between α_3_ and α_2_ in study 2	1.87 (6.4)
E_max_, Study 1	E_max_ for study 1	2.65 (13)
E_max_, Study 2	E_max_ for study 2	3.55 (30)
EC_50_	Concentration (ng/mL) reaching half of E_max_	0.365 (66)
k_e0_	Equilibrium rate constant for response in study 1 and placebo effect (h^-1^)	0.00217 (7.6)
θ_Emax_, race	Coefficient on E_max_ in Black/AA	1.43 (7.2)
θ_Emax_, fentanyl, no injection	Coefficient on E_max_ for fentanyl use ≥2 times/day with no injection use at baseline	0.736 (12)
θ_Emax_, fentanyl+injection	Coefficient on E_max_ for fentanyl use ≥2 times/day and injection use at baseline	0.198 (115)
θ_Placebo_	Placebo maximal effect	3.73 (7.4)
IIV α	SD	1.71 (8.5)
IIV E_max_	CV%	53.0 (29)
IIV EC_50_	CV%	127 (67)
IIV k_e0_	CV%	190 (4.4)

AA, African americans; CV%, Coefficient of variation expressed as a percentage; IIV, Interindividual variability; SD, standard deviation; RSE, Relative standard error. ‘Injection use’ means that injection was reported as the main route of opioid use at baseline

### 3.5 Dropout model

The dropout dataset included information on study discontinuation/completion from 1,071 participants (489 in Study 1 and 582 in Study 2). The higher dropout observed at the beginning of both studies indicated a decrease in the hazard of dropout over time (Supplementary Figure 4). There were no differences in treatment retention between BUP-XR maintenance doses in either study (Supplementary Figure 4).

**FIGURE 5:**
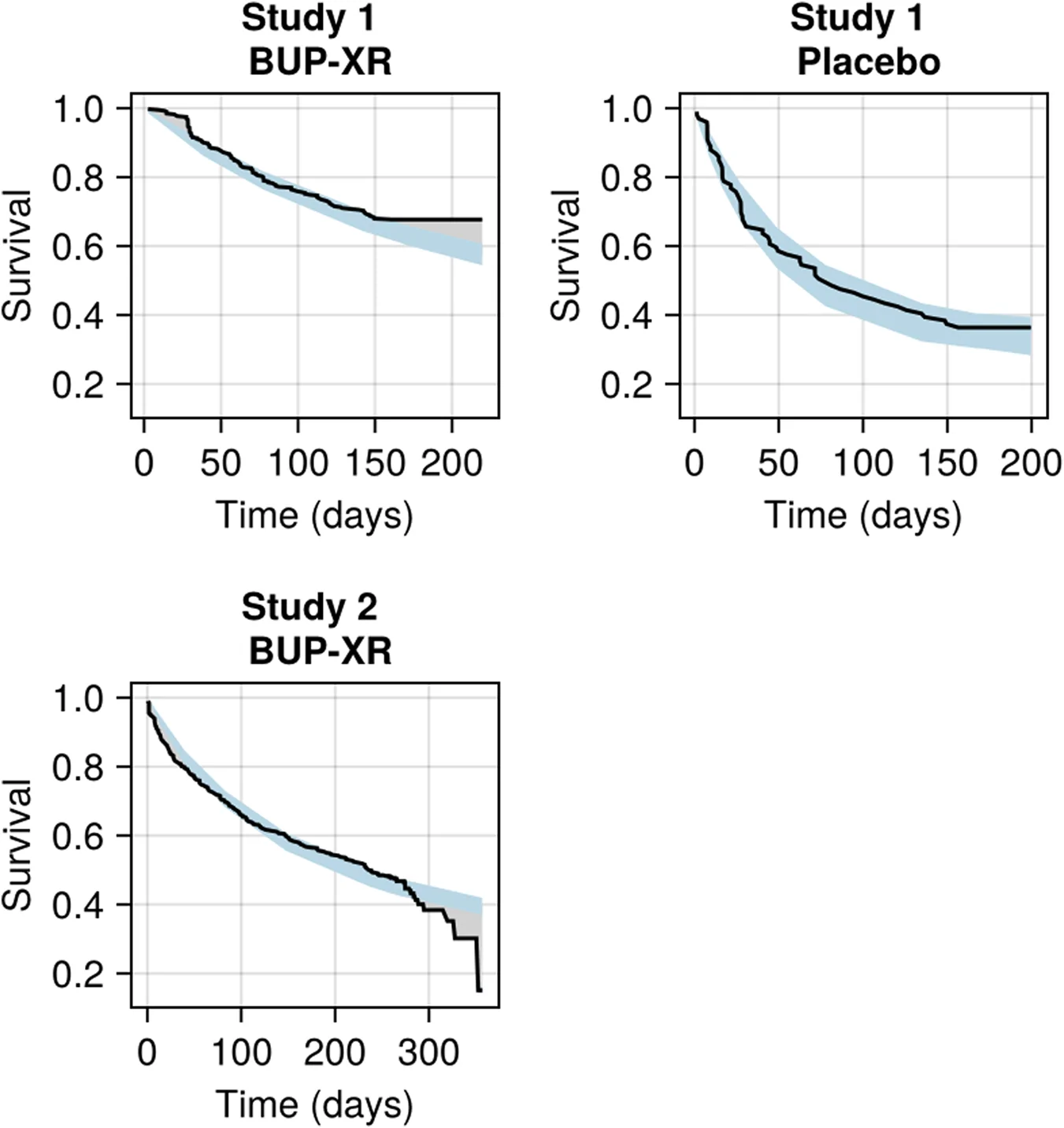
Observed versus Predicted Treatment Retention for Extended-Release Buprenorphine (BUP-XR) Treatments Arms in Study 2 and Study 1, and Placebo Arm in Study 1. Lines represent the survival probability over time derived from the observed data; the blue shaded regions delineate the 95% confidence region predicted by the model; the grey shaded regions highlight deviations from the model.

Time-to-event analysis was conducted to model treatment retention over time and to assess the relationship with past opioid use and opioid craving under a missing at random assumption. Treatment retention data from the combined analysis of both studies were well described by a Gompertz model that accounted for the higher dropout rate observed at the beginning of each study (Figure 5). Separate model parameters were estimated for patients receiving placebo in Study 1.

Opioid craving was identified as a major predictor of treatment discontinuation with a 2-fold higher hazard of dropout when craving exceeded 20 on the OC-VAS. No major difference in treatment retention was observed for craving values below 20. Additionally, the hazard for dropout was found to decrease with age and was 34% lower in Black/African American participants. The effect of age was less pronounced in BUP-XR treatment patients than in placebo patients, with estimates of −0.581 and −1.70, respectively. The final model parameter estimates are provided in Table 4. Model evaluation using visual predictive checks indicated good agreement between observed and predicted data when stratified by study and active versus placebo treatment (Figure 5), race (Supplementary Figure 5) and age (Supplementary Figure 6). Final model equations are included in the Supplemental Material.

**TABLE 4:** Parameter estimates for the final treatment retention model.

Parameter	Description	Estimate (RSE%)
β_0_ BUP-XR	Baseline hazard with BUP-XR	0.00332 (12)
β_0_ Placebo	Baseline hazard with placebo, study 1	0.00860 (23)
k_e_ BUP-XR	Rate for exponential decrease in hazard over time with BUP-XR (day^-1^)	0.00271 (27)
k_e_ Placebo	Rate for exponential decrease in hazard over time with placebo (day^-1^)	0.00906 (36)
β_1_ craving=1–5	Coefficient for craving = 1–5	0.897 (13)
β_1_ craving=6–20	Coefficient for craving = 6–20	1.04 (15)
β_1_ craving >-20	Coefficient for craving >20	2.28 (12)
β_2_ age, BUP-XR	Age effect (power model)	−0.581 (32)
β_2_ age, Placebo	Age effect (power model) in placebo patients	−1.70 (27)
β_3_ race	Coefficient for race (black/African american)	0.663 (13)

## 4 Discussion

To our knowledge, this is the first buprenorphine exposure-response analysis in the context of changing synthetic opioid use and polysubstance use patterns among patients with moderate to severe OUD in the US. This integrated analysis included two large clinical trials: Study 1, conducted in 2015–2016 prior to the widespread emergence of fentanyl, and Study 2, conducted in 2021–2024, in which most participants reported fentanyl use, half reported fentanyl use daily, and 95% reported polysubstance use. Our findings offer important insights into determinants of treatment response and build upon a previous exposure-response analysis that was solely based on Study 1 (Laffont et al., 2022).

### 4.1 Opioid use

A main finding from our opioid use exposure-response analysis was an estimate of 1 ng/mL for buprenorphine EC_50_ (concentration associated with half of maximal effect). This value is very close to the previous estimate of 1.2 ng/mL based on Study 1 alone (Laffont et al., 2022) and corresponds to approximately 50% brain μ-opioid receptor occupancy (Nasser et al., 2014). An EC_50_ of 1 ng/mL implies that buprenorphine plasma concentrations of 2–3 ng/mL (delivered by 100 mg BUP-XR monthly) would achieve near-maximal benefit on opioid abstinence in most patients. This aligns with clinical observations in both studies, showing no meaningful differences in abstinence between the 100-mg and 300-mg maintenance dose groups at the population level (Shiwach et al., 2025b).

Some subgroups of patients, however, showed a higher buprenorphine EC_50_. Strikingly, an 8-fold higher EC_50_ was estimated in patients reporting daily fentanyl use in combination with amphetamines/methamphetamines at baseline, which suggests these patients might benefit from higher buprenorphine exposure. These modeling results are consistent with clinical observations from Study 2, in which opioid abstinence rates were consistently lower in this group of patients when treated with the 100-mg maintenance dose but comparable to the rest of the population when treated with the 300-mg maintenance dose as concentrations reached steady-state (Figure 3). These results refine previous post-hoc analyses of Study 2 indicating that frequent fentanyl users (i.e., using fentanyl daily and/or ≥14x/week) were more likely to benefit from the 300-mg maintenance dose (Shiwach et al., 2025b). Although an overall fentanyl effect was observed in our modeling study, the interaction with the use of amphetamines/methamphetamines appeared to be a key determinant of treatment response. Identification of this subgroup (about 30% of participants in Study 2) is clinically relevant considering the fourth wave of the opioid epidemic and the risks associated with the co-use of opioids and stimulants, including increases in opioid overdose fatalities and poorer treatment outcomes (Frost et al., 2021; Russell et al., 2023; Coles et al., 2025). A key finding of our analysis is that outcomes in this patient subgroup may be improved through higher buprenorphine levels such as those delivered by BUP-XR 300 mg monthly.

Another finding was a slightly higher EC_50_ in patients reporting injection as the main route of opioid administration. This finding aligns with the previous exposure-response analysis (Laffont et al., 2022; Greenwald, et al., 2023), although the increase in EC_50_ was less than previously estimated (1.74-fold vs. 3.67-fold) and seemed mainly driven by Study 1 data. In Study 2, no significant differences in opioid abstinence were reported between injecting opioid users and non-injecting opioid users (Shiwach et al., 2025b). A potential explanation for these disparate results is that high-risk opioid use may no longer be associated with intravenous injection. Across the two studies, the proportion of participants reporting injection as their main route of use decreased from 43.8% in Study 1 to 29.2% in Study 2, whereas the use of smoking increased from 6.5% to 41.2% (snorting use was similar). These trends are consistent with changing modalities of opioid use in the US (Karandinos et al., 2024) and reports of decreasing overdose deaths associated with opioid injection alongside increasing overdose deaths associated with opioid smoking (Tanz et al., 2024). Smoking and snorting represent more practical and safer routes than intravenous injection for frequent fentanyl use as they reduce injection-related harms (infection, transmission of blood-borne infectious diseases; Megerian et al., 2024) while still allowing rapid absorption and penetration into the brain (Bird et al., 2023).

A few additional covariates with smaller effects were identified for opioid use, including race and employment at baseline. Surprisingly, the *OPRD1* genotype assessing SNP rs678849 was not retained as a significant covariate on EC_50_ whereas significant effects were previously estimated for Study 1 (71% and 94% decreases in EC_50_ with CT and TT genotypes, respectively, relative to CC genotype). Although all genotypes were well represented in Study 2 at frequencies similar to Study 1, the most recent data did not replicate previous findings. It is noteworthy that former trials identified this variant as a predictor of buprenorphine treatment response in Black/African Americans, not European Americans (Crist et al., 2013; Crist et al., 2019). Thus, more contemporary drug-use patterns observed in Study 2 (i.e., greater smoking than injection, fentanyl and stimulant use) may overshadow any genotype effect that was identified in Study 1. It is also possible that these changing drug-use patterns are associated with race, which would thereby reduce the explanatory power of genotype. Thus, future research should investigate race-stratified effects.

Importantly, participants in Study 2 required longer time on buprenorphine treatment to reach maximal effects on opioid abstinence, which was unsurprising given the high-risk population enrolled. A major takeaway from a previous analysis of 18-month treatment data with BUP-XR is that patients initially categorized as slow-responders ultimately reached abstinence levels that were comparable to the rest of the population (Rutrick et al., 2023). Based on these observations, an effect compartment was included in the opioid use model to adequately describe the continuous improvement of patients in Study 2, which greatly improved the model performance. The average estimated time to achieve optimal abstinence outcomes in Study 2 high-risk population was 6 months; however, this 6-month duration was associated with high inter-patient variability, which could not be explained by any of the covariates investigated. It is equally important to distinguish long-term treatment effects (abstinence) from short-term treatment effects (such as the decrease in opioid-use frequency). In Study 2, self-reported opioid use dropped dramatically from 43 times/week at baseline to just 2.6 times/week by the second week of treatment, and these harm-reduction benefits were sustained throughout the study (Shiwach et al., 2025b). These results highlight that early non-abstinence should not be interpreted as treatment failure and that discontinuing treatment early because a patient continues to use fentanyl or other opioids intermittently may not allow sufficient time for the medication to achieve its full therapeutic effect.

### 4.2 Opioid craving

In the opioid craving exposure-response model, the estimated buprenorphine EC_50_ (0.4 ng/mL) was noticeably lower than the previous estimate (2.45 ng/mL, Laffont et al., 2022). In Study 2, administration of the first two injections of 300-mg BUP-XR 1 week apart led to rapid attainment of buprenorphine plasma concentrations ≥2 ng/mL and concurrent rapid decrease in OC-VAS scores. Accordingly, the dataset may have contained insufficient observations at lower buprenorphine concentrations to adequately characterize the EC_50_, which would explain the low precision of estimation (66%). Additionally, a placebo effect was included to improve description of placebo data, which could also have affected EC_50_ estimation. Like the opioid-use model, the model for opioid craving incorporated an effect compartment for Study 2 predicting maximum craving response after 2 months on average.

Based on observed data and the lack of significant covariate effects on EC_50_, there were no apparent benefits of the 300-mg maintenance dose relative to the 100-mg maintenance dose on craving. Maximum treatment response (E_max_) was 26% lower in participants reporting frequent fentanyl use at baseline (≥2 times/day; 44% of Study 2 population) and 80% lower in participants reporting both frequent fentanyl use (≥2 times/day) *and* injection use at baseline (6% of Study 2 population). These lower maximum response estimates are consistent with the overall higher proportion of patients reporting clinically significant craving (>20 mm) in Study 2 compared to Study 1 and align with previous reports that fentanyl could generate more severe craving (Casey et al., 2025).

A 43% higher maximum treatment effect on craving was estimated in Black/African Americans. Rather than showing higher efficacy of buprenorphine treatment, this finding may instead reflect cultural determinants of symptom reporting, whereby patients may underrepresent the actual severity of their cravings (Brown et al., 2010; Ross et al., 2024). For example, Brown et al. (2010) reported higher objective and subjective withdrawal scores and greater opioid craving in non-Hispanic Caucasians compared to minority participants at the time of buprenorphine induction. Hence, further research is needed to understand why lower craving scores were reported by Black/African American patients in the present study.

### 4.3 Treatment retention

The time-to-event analysis confirmed elevated craving (>20 mm on OC-VAS) as a key predictor of dropout. The proportion of patients with craving scores >20 was 20% in Study 2 versus 6% in Study 1. Once the effect of craving was accounted for in the model, the same model parameters could describe treatment retention in both studies despite apparent differences in retention rate, which underscores the robustness of the model.

In line with previous findings (Laffont et al., 2022), a 34% lower dropout rate was estimated in Black/African Americans compared to whites people/person(s). The reasons for this finding are unclear and may be confounded with other factors. Modeling showed a higher dropout rate with younger age, and this age effect was more pronounced in the placebo group than in BUP-XR treatment groups. Age was previously reported as an important predictor of dropout in several buprenorphine clinical studies where younger age was associated with shorter retention (Hser et al., 2014; Schuman-Olivier et al., 2014; Marcovitz et al., 2016).

Frequent fentanyl use and/or amphetamine/methamphetamine use at baseline did not directly impact treatment retention in this analysis. Although mixed findings have been reported in the literature, a few studies have described lower treatment retention in patients reporting stimulant use (Frost et al., 2021; Coles et al., 2025). However, all these previous studies were conducted with daily transmucosal buprenorphine, and it is possible that use of a long-acting injectable, such as BUP-XR, reduced the overall probability of treatment discontinuation and, thus, any differential association with stimulant use.

### 4.4 Strengths and limitations

This work presents several strengths. The analysis included two large clinical studies with longitudinal measurements over 6–11 months evaluating low and high maintenance doses of BUP-XR. Studies were conducted during distinct time periods encompassing the past decade, allowing for evaluation of buprenorphine exposure-response amid epidemiological changes in synthetic opioid use patterns and polysubstance use. Although self-reported substance use may be subject to recall bias or underreporting, concordance analyses demonstrated good agreement between self-reported use and urine drug screen results for opioids and other substances in Study 2, such as amphetamines/methamphetamines and cocaine (Supplementary Table 2).

Some limitations should also be acknowledged. First, fentanyl use was not assessed in Study 1 and frequent fentanyl use (daily or ≥2x/day) was assumed to be absent. Considering the timing of Study 1 (2015–2016) and the limited availability of illicitly manufactured fentanyl during this period, this assumption appears reasonable. Second, Study 2 enrolled a high-risk population using opioids ≥5 days per week, which may not fully represent today’s patient population in the US. However, the combination of Studies 1 and 2 may be considered to cover a broad spectrum of patient severity. Finally, while percentages of polysubstance use (95%) and amphetamine/methamphetamine use (60%) aligned with recent studies (El Bassel et al., 2026), the proportion of cocaine users was relatively small with only 9.8% reporting daily fentanyl use with cocaine at baseline. This may reflect the geographical distribution of study sites, as only 4 of the 28 sites were in the Northeastern US where fentanyl use with cocaine is predominant (Friedman and Shover, 2023). The limited number of participants reporting daily fentanyl use with cocaine may have limited evaluation of the impact of cocaine co-use in this analysis.

## 5 Conclusion

This integrated exposure-response analysis of two large clinical studies spanning the pre-fentanyl and fentanyl eras provides important insights into determinants of buprenorphine treatment response. Contemporary buprenorphine treatment may be underdosed or inadequately sustained in fentanyl-exposed polydrug populations. The present analysis demonstrates that patients with daily fentanyl use and amphetamine/methamphetamine co-use may require substantially higher buprenorphine exposure to achieve optimal outcomes. Additionally, levels of opioid abstinence were lower in the most recent study. This difference, reflecting the greater severity of the enrolled population, appeared to be driven by a higher proportion of slow responders who required longer time on treatment. This is consistent with emerging evidence that sustained treatment engagement is needed to optimize long-term outcomes (Hayes et al., 2026). Altogether, these findings provide clinically meaningful guidance for optimizing buprenorphine treatment and advancing understanding of patient journey in the era of synthetic opioids and stimulant co-use.

## Data Availability

The datasets used for analysis are not readily available because patient-related data were generated as part of clinical trials and may be subject to patient confidentiality. Data are available from the authors upon reasonable request. All requests for raw and analysed data will be promptly reviewed by the corresponding author or delegate to verify if the request is subject to any confidentiality obligations. Any data that can be shared will be released via a data use agreement. Requests to access the datasets should be directed to Celine Laffont celine.laffont@indivior.com.
