# Supplementary material for: Optimal buprenorphine exposure to treat patients with opioid use disorder in the era of fentanyl and polysubstance use
Source: Front Pharmacol. 2026 Sep 8;17:1839067. doi: 10.3389/fphar.2026.1839067 (PMC13593607; doi:10.3389/fphar.2026.1839067)
Supplement: Supplementary file 1 [file Supplementaryfile1.docx]

Supplemental Table 1:	Description of Studies Included in Exposure-Response Integrated Analyses

| Study  Number/Time of Conduct | Study Design | Description | Population | PK Assessments | PD Assessments  of Interest |
| --- | --- | --- | --- | --- | --- |
| 1  2015-2016 | Randomised, DB, placebo-controlled,  24-week efficacy, safety and tolerability study | Participants were inducted using TM BUP for 3 days, followed by 4- to 11-day dose adjustment on TM BUP doses ranging from 8 to 24 mg/day.  300/300 mg active group:  300 mg for 6 injections separated by 28 ± 2 days (N=201)  300/100 mg active group:  300 mg for the first 2 injections followed by 100 mg for subsequent 4 injections separated by 28 ± 2 days (N=203)  Placebo group: volume-matched to 300/300 mg group or 300/100 mg group (N=100) | Treatment-seeking participants with moderate to severe OUD (based on DSM-5)  Males and females  18-65 years of age  BMI ≥18.0 to ≤35.0 kg/m^2^ | Run-in phase on Day -1:  Pre-dose and 1-2 hours post-dose  DB treatment phase: Weekly PK sampling, with additional samples taken at 4 hours after each injection | UDS/self-reports assessed at Screening and weekly visits following each SC injection, as well as at the safety follow-up visit on Day 197. UDS were also performed at 24 hours after each injection.  Opioid craving scores (OC-VAS) assessed at the same times as UDS (except for the follow-up safety visit), with additional measures collected during the run-in period. Only current craving was assessed. |
| **2**  2021-2024 | Randomised, DB 37-week efficacy study | OL induction phase (Weeks 1-6):  Standard of care induction with TM BUP for ≥ 7 days followed by injection of 300 mg BUP-XR vs. rapid induction with 1 dose of 4 mg TM BUP followed by injection of 300 mg BUP-XR.  The second injection of 300 mg BUP-XR was given at 1 week (+4 days) after the first injection.  DB maintenance phase (Weeks 6-38):  Randomisation at Week 6 to maintenances doses of 100 mg or 300 mg BUP-XR for a total of 8 additional injections. Maintenance doses were separated by 28 (-2/+4) days.  785 participants were randomised in the OL substudy; 603 received the first injection of BUP-XR; 436 were randomised to DB maintenance treatment (219 to 100 mg monthly, and 217 to 300 mg monthly); 262 completed the study. | Treatment-seeking participants with moderate to severe OUD (based on DSM-5) and high-‑risk opioid use (IV use or use of high doses of opioids or any dose of highly potent synthetic opioids [fentanyl and analogues excluding transdermal patches])  Males and females  At least 18 years of age  No BMI inclusion criteria | PK samples were taken at each scheduled visit (weekly from Week 1 to Week 10, monthly thereafter until Week 34, then on Weeks 36 and 38).  On the day of the first SC injection, 2 PK samples were collected: one at pre-dose and one at 2 hours post dose. | UDS/self-reports assessed at Screening and at each scheduled visit (weekly from Week 1 to Week 10, monthly thereafter until Week 34, then on Weeks 36 and 38).  Additional visits (2 weeks ± 7 days post each injection) were scheduled at random by the Investigator between Injections 4 to 10 to collect UDS/self-reports.  Opioid craving scores (OC-VAS) assessed at Screening and at each scheduled visit (weekly from Week 1 to Week 10, monthly thereafter until Week 34, then on Weeks 36 and 38). Both current craving and worst craving over the past week were assessed. |
| BMI: body mass index; BUP: buprenorphine; BUP-XR: extended-release buprenorphine; DB: double-blind; DSM-5: Diagnostic and Statistical Manual of Mental Disorders, 5th Edition; IV: intravenous; N: sample size; OC-VAS: opioid craving visual analog scale; OL: open-label; OUD: opioid use disorder; PD: pharmacodynamic; PK: pharmacokinetic; TM: transmucosal; UDS: urine drug screen | | | | | |

Supplemental Table 2:	Concordance between Self-reported Use and Urine Drug Screen at Screening in Study 2

|  |  | **Negative TLFB** | **Positive TLFB** |
| --- | --- | --- | --- |
| **Amphetamine/Methamphetamine** | | | |
|  | Negative UDS | 164 | 17 |
|  | Positive UDS | 37 | 306 |
| **Cocaine** | | | |
|  | Negative UDS | 340 | 32 |
|  | Positive UDS | 31 | 97 |
| **Opioids** | | | |
|  | Missing UDS | 0 | 2 |
|  | Negative UDS | 0 | 10 |
|  | Positive UDS | 1 | 487 |

**Opioid Use Model Equations**

The probability of negative opioid use was modelled using mixed-effects logistic regression as follows:

$$logit\left[ P\left( Y_{ij}=0 \right) \right]=\alpha+fC+\eta_{i}$$

$$P\left( Y_{ij}=0 \right)=\frac{e^{\left( \alpha+fC+\eta_{i} \right)}}{1+e^{\left( \alpha+fC+\eta_{i} \right)}}$$

$$P\left( Y_{ij}=1 \right) =1-P\left( Y_{ij}=0 \right)$$

Where *Y_ij_* is the observation for subject *i* at time t_ij_, α is the intercept at the logit level, $fC$(E_max_ model) is a function of BUP exposure, and $\eta_{i}$ is the subject-specific random effect associated with the intercept following a normal distribution.

Interindividual variability was additionally tested on E_max_ model parameters as well as k_e0_ (equilibration rate constant for effect compartment in Study 2) assuming log-normal distributions.

**Opioid Craving Model Equations**

Categorical opioid craving was evaluated using mixed-effects logistic regression modeling for ordinal data. If *Y_ij_* denotes the observation in subject *i* at time *t*_i_*_j_*, the probability for *Y_ij_* to be less than or equal to *m* (m=1, 2, or 3) is expressed as:

$$P\left( Y_{ij}\leq m \right) = \frac{e^{\left( \alpha_{m}+fC+\eta_{i} \right)}}{1+e^{\left( \alpha_{m}+fC+\eta_{i} \right)}}$$

and

$$logit\left[ P\left( Y_{ij}\leq m \right) \right]=\alpha_{m}+fC+\eta_{i}$$

Where $\alpha_{m}$ is the corresponding intercept, $fC$is the BUP effect (E_max_ model), and $\eta_{i}$ is the subject-specific random effect associated with the intercept following a normal distribution. To account for the ordinal nature of the data, $\alpha_{1}<\alpha_{2}<\alpha_{3}$.

The probability of observing a score in each category was formulated as:

$$P\left( Y_{ij}=1 \right)=P\left( Y_{ij}\leq1 \right)$$

$$P\left( Y_{ij}=2 \right)=P\left( Y_{ij}\leq2 \right)-P\left( Y_{ij}\leq1 \right)$$

$$P\left( Y_{ij}=3 \right)=P\left( Y_{ij}\leq3 \right)-P\left( Y_{ij}\leq2 \right)$$

$$P\left( Y_{ij}=4 \right)=1-P\left( Y_{ij}\leq3 \right)$$

Adequate description of placebo data required the addition of a placebo model. Hence, the final model was as follows:

$$logit\left[ P\left( Y_{ij}\leq m \right) \right]=\alpha_{m}+fC+{f_{placebo}+\eta}_{i}$$

Where:

$$f_{placebo}= \theta_{placebo}\times\left( 1-e^{-ke0\times t} \right)$$

Interindividual variability was tested on E_max_ model parameters as well as k_e0_ assuming log-normal distributions.

**Treatment Retention Model Equations**

In the prior analysis of Study 1, a Gompertz hazard model best described the dropout of study participants:

$$hazard=\beta_{0} \times exp\left( -ke\times t \right)$$

where $\beta_{0}$ is the baseline hazard i, and $ke$ is the rate constant for the exponential decrease in hazard over time.

Survival to a given time ($t_{i}$) was calculated for each subject $i$ as follows:

$$Survival(t_{i})=exp\left( -\int_{0}^{t_{i}} hazard(t)dt \right)$$

The final model was as follows for active treatment groups of both studies:

$$hazard=\beta_{0 BUP-XR}\times\beta_{1}^{Craving LOCF}\times\left( \frac{Age}{40} \right)^{\beta_{2 BUP-XR}}\times\beta_{3}^{RACE}\times exp\left( -{ke}_{BUP-XR}\times t \right)$$

The final model was as follows for the placebo group of Study 1:

$$hazard=\beta_{0 Placebo}\times\beta_{1}^{Craving LOCF}\times\left( \frac{Age}{40} \right)^{\beta_{2 Placebo}}\times\beta_{3}^{RACE}\times exp\left( -{ke}_{Placebo}\times t \right)$$

**300/300 mg**

**300/100 mg**

Supplemental Figure 1. Study 2 Visual Predictive Checks for Existing Population Pharmacokinetic Models. Lines represent the median and 5^th^/95^th^ percentiles of the observed data; shaded regions delineate the confidence intervals of the 5^th^, 50^th^, and 95^th^ percentiles calculated from simulated data. BUP: buprenorphine.

Model 95%CI

Observed

Predicted

Supplemental Figure 2. Visual Predictive Checks for the Final Opioid Use Model. Data binning was performed to generate visual predictive checks. Model predicted and observed data calculated for each bin were plotted against the average of the time points defining each bin. Solid lines represent observed percentages with their 95% confidence intervals (CIs) shown as error bars; dashed lines represent predicted percentages with their 95% CIs (shaded regions).

Supplemental Figure 3. Visual Predictive Checks for the Opioid Craving Model Data binning was performed to generate visual predictive checks. Model predicted and observed data calculated for each bin were plotted against the average of the time points defining each bin. Solid lines represent observed percentages with their 95% confidence intervals (CIs) shown as error bars; dashed lines represent predicted percentages with their 95% CIs (shaded regions).

**Supplemental Figure 4.** Kaplan-Meier Plots by Study and Treatment Arm**.** BUP-XR: extended-release buprenorphine; OL: open label, which includes participants that discontinued prior to randomization on Week 6.

Supplemental Figure 5. Visual Predictive Checks for the Final Dropout Model Per Study, Treatment Group, and Race. Lines represent the survival probability over time derived from the observed data; the blue shaded regions delineate the 95% confidence region predicted by the model; the grey shaded regions highlight deviations from the model. AA: African American; BUP-XR: extended-release buprenorphine; CI: confidence interval

**Supplemental Figure 6.** Visual Predictive Checks for the Final Dropout Model Per Study, Treatment Group, and Age Group. Lines represent the survival probability over time derived from the observed data; the blue shaded regions delineate the 95% confidence region predicted by the model; the grey shaded regions highlight deviations from the model. BUP-XR: extended-release buprenorphine; CI: confidence interval
